# Self-management behaviors do not affect remission but mediate between mental health and disease outcomes in a longitudinal study of rheumatoid arthritis

**DOI:** 10.1007/s00296-024-05761-8

**Published:** 2025-01-17

**Authors:** Melissa Sweeney, Lewis Carpenter, Savia de Souza, Emma Caton, James Galloway, Andrew Cope, Mark Yates, Elena Nikiphorou, Sam Norton

**Affiliations:** 1 Health Psychology Section, Institute of Psychiatry, Psychology and Neuroscience, King’s College London, London, UK; 2 Centre for Rheumatic Diseases, King’s College London, London, UK

**Keywords:** Depression, Anxiety, Arthritis, Rheumatoid, Self-management

## Abstract

Mental health has been shown to impact rheumatoid arthritis (RA) outcomes and is associated with self-management behaviors. The extent to which mental health impacts outcomes via different self-management behaviours has not been thoroughly investigated. Adult RA patients who were starting a new medication or dosage were recruited to a prospective cohort with follow-ups at 3 and 12-months covering clinical and patient-reported outcomes. The longitudinal relationships between mental health, self-management behaviors (diet, physical activity, sleep, smoking, alcohol, and medication nonadherence), disease outcome, and function were assessed. Self-management behaviors were considered mediators of mental health at baseline on outcomes at 3 and 12 months. Depression did not worsen the odds of EULAR response for the total PHQ at 3 months (OR = 0.96, *p* = 0.36) or 12 months (OR = 0.99, *p* = 0.99) nor for the categorical PHQ at 3 months (OR = 0.64, *p* = 0.34) or 12 months (OR = 0.67, *p* = 0.44). Anxiety also did not worsen the odds of EULAR response for the total GAD at 3 months (OR = 0.98, *p* = 0.76) or 12 months (OR1.04, 0.53) nor for the categorical GAD at 3 months (OR = 0.99, *p* = 0.99) or 12 months (OR = 0.94, *p* = 0.75). However, depression was associated with the DAS-28 at 3 months (b = 0.22, *p* = 0.04). Among the self-management behaviors, insomnia was found to be a significant mediator between depression and the WSAS (b = 0.08, *p* = 0.03) as well as anxiety and the WSAS (b = 0.07, *p* = 0.03). Alcohol was also a significant mediator between depression and the DAS-28 (b = 0.21, *p* = 0.04). Mental health was associated with worse quality of life and disease outcomes, but not EULAR response. Self-management behaviors were associated with disease outcomes and mental health.

**Supplementary Information:**

The online version contains supplementary material available at 10.1007/s00296-024-05761-8.

## Introduction

Rheumatoid Arthritis (RA) is a chronic autoimmune disorder affecting approximately 0.7% of the UK population [1] where inflammation leads to joint synovitis, pain, and fatigue. The condition has a substantial impact on quality of life [2–4]. Around 1 in 4 people experience high levels of psychological distress at some point during the disease course [5, 6]. Biological therapies appear to not fully address the somatic aspects of disease, such as pain and fatigue, though targeted synthetic disease-modifying antirheumatic drugs (DMARDs) may have a better outcome [7, 8]. For patients whose response to treatment is unsatisfactory, it is unclear the extent to which continuing elevated disease activity is driven by inflammatory factors, or other mechanisms, such as behavioral factors linked to self-management [9, 10]. Treatment response, particularly on somatic symptoms, appears to be influenced by mental health [11–13]. By improving understanding of the relationship between mental health, self-management, and disease outcomes, patients can be better supported at earlier stages to prevent downstream negative consequences on RA outcomes. Additionally, more detailed clarity on the roles of specific health behaviors can better inform patients with accurate information for their self-management.

Poor mental health has been linked to a reduced ability to self-manage physical health problems that often involve multiple complex behaviors, and thus may influence disease outcomes predominantly via these pathways. Effective self-management is recommended for RA and improves outcomes such as pain, physical functioning, and self-management behaviors [14]. The EULAR recommendations highlight the importance of screening and support in skills for self-management. The importance of this support is evidenced by patients demonstrating poor self-management when mental health is poor [15]. Currently no studies have directly investigated whether self-management behaviours mediate the effect of mental health on disease outcomes.

This study aims to investigate the impact of mental health on disease outcomes and self-management-related factors in RA. Specifically, this study will further investigate which self-management behaviors are most influential in mediating the complex relationships between mental health and disease outcomes.

## Methods

### Design & recruitment

The Patient Reported Outcomes in Persistent Rheumatoid Arthritis (PROsPer-RA) study is a prospective cohort of patients with confirmed RA undergoing a treatment change with follow-up at 3 and 12-months from 2019 to 2022. A Patient Research Partner with established RA (SdS) was involved with the study design. Participants were recruited through nine rheumatology clinics around the United Kingdom. Data was collected at baseline, 3 months, and 12 months in clinic and by mail. Data was collected at clinical appointments by clinical nurses. The surveys were self-reported and completed by mail. Eligibility criteria were: aged 16 and above, confirmed diagnosis of RA, switching to a different DMARD of any type or a dose increase due to lack of efficacy. The exclusion criteria were: under 16 years old, primary diagnosis of other rheumatic disease, established disease with the same treatment for over one year, severe comorbidities impacting ability to participate, and inability to provide written informed consent or complete questionnaires.

### Measures

At each timepoint there was a patient self-report questionnaire covering demographics (using a fixed set of categories), RA disease symptoms and activity, mental health, quality of life, self-management behaviors, and views about health. Clinical data were also collected at each timepoint covering disease activity, tender and swollen joints, inflammatory markers, medications, comorbidities, and allied health professional treatments.

#### Arthritis symptoms and disease activity

The DAS-28 (disease activity score based on a 28 joint count) was used as an outcome measure for disease activity. It is considered the gold standard for disease activity according to the ACR and EULAR [16].

Visual Analog Scales (VAS) were used for pain and fatigue. VAS are considered appropriate for measuring intensity of experiences [17]. For example, the fatigue VAS has an intraclass correlation coefficient of 0.74 [18]. Similarly, the pain VAS correlates well (*r* = 0.80–0.96) with other pain scales [19].

#### Mental health

The mental health measures used in this study were the Personal Health Questionnaire (PHQ-9) and Generalized Anxiety Disorder assessment (GAD-7). Both have demonstrated acceptable diagnostic accuracy and reliability in validation studies [20, 21]. The PHQ-9 has an 88% sensitivity and 89% specificity for major depression and was shown to be valid in comparison to an interview by a health professional and has good internal reliability (Cronbach’s alpha = 0.86) [20]. The GAD-7 also has high sensitivity (89%) and specificity (82%) for Generalized Anxiety Disorder [21]. It also shows good internal consistency (Cronbach’s alpha = 0.92) and test-retest reliability (intraclass correlation = 0.83) [21].

#### Behavioral factors

The Insomnia Severity Index (ISI) has been shown to be one of the top-ranked scales for insomnia in RA patients in terms of feasibility [22]. It also shows good validity on each subcomponent compared to a sleep diary (*r* = 0.32 to 0.55) [23]. The brevity and self-report aspects of the ISI were limitations that were weighed in relation to feasibility in terms of the length and cost.

Body Mass Index (BMI) was also included because it is related with both diet and physical activity, but due to the barriers RA patients may face in engaging in physical activity, the level of motivation for physical activity may not necessarily translate directly into corresponding physical activity levels [24].

The shortened version of the International Physical Activity Questionnaire (IPAQ) was used for brevity [25]. The IPAQ is one of the most commonly used measures of physical activity in RA [24] and has high reliability (*r* = 0.80) [26].

The Alcohol Use Disorders Identification Test (AUDIT) is commonly used in RA research to measure alcohol use [27, 28]. It has shown reliability above 0.80 across numerous studies [29]. It has a sensitivity of 70–85% and specificity of 73–94%, depending on the cutoff score used [30]. Thus, the AUDIT was used because it accommodates a balance between feasibility and accuracy.

Since diet was not the main focus of the PROsPER-RA questionnaire, it was limited to two researcher-designed questions. Shortened questionnaires have been shown to have better feasibility but less accuracy compared with longer questionnaires [31]. The two questions used in the PROsPER-RA study were summed to give a total diet score. The questions used were: “During the last 7 days, on how many days have you consumed 5 or more portions of fruit and/or vegetables?” and “During the last 7 days, on average how many portions of fruit and/or vegetables did you consume per day?”.

One researcher-designed question was also used for smoking. Determining smoking status by self-report has been found to be accurate in research studies [32, 33]. The participants were asked if they were current or past smokers.

Lastly, medication adherence was also determined by a self-report researcher-designed question: “Do you always take your medication at the specified time?” with answer options ranging from “Never” to “Always”. Patient self-report is the most commonly used method for assessing medication adherence [34]. Self-report estimation of medication adherence has been found to have varying correlation levels with objective measures such as pill counting, but overall validity studies have determined that estimation can perform equally well compared with count-based measures [34].

#### Quality of life

The work and social adjustment scale (WSAS) was used to measure quality of life [35]. It has shown good reliability with Cronbach’s alpha = 0.93 [36]. Its main limitation is that it has not been specifically evaluated for use in Rheumatoid Arthritis populations so this could lead to inaccuracies in that it could be less valid in certain populations. However, it has been evaluated in a variety of chronic illnesses more generally [37].

### Statistical analysis

The demographics for three different potential samples were initially calculated to compare those with missing data longitudinally with those with sufficient data to be included in the analysis, to identify potential bias from missing data since there were multiple follow-ups and both clinical and patient data collections at each wave.

The overall sample was the initial sample for which demographics were calculated (Table 1). Since the initial target sample size was 400, a power analysis was conducted to determine if the sample size was sufficient to detect reasonable effects for the planned tests of mediation. These were not originally planned when the sample size was selected. For the sample size of 209, there was 80% power to detect small- to medium-sized mediation effects. Specifically, assuming small standardized effects of predictor on mediator (A path) and mediator on outcome (B path), respectively, and subsequently a small mediated effect using the product of coefficients approach (AxB = 0.16).

The analysis of data is presented in several overarching sections as follows: Changes in outcomes over time, correlations between variables of interest at baseline and outcomes over time, EULAR response, and mediation models. Further details of the mediations is also presented in the supplementary material.

The sample of anyone who returned baseline plus any longitudinal patient data was compared with those who only completed the baseline (Table S2). After selecting the sample, the means at baseline for key variables was calculated. Violin plots were also generated for the means. A correlation matrix was created between self-management behaviors and the remaining variables. Next, odds ratios were calculated for EULAR response criteria to determine improvement in DAS-28 scores.

**Fig. 1:**
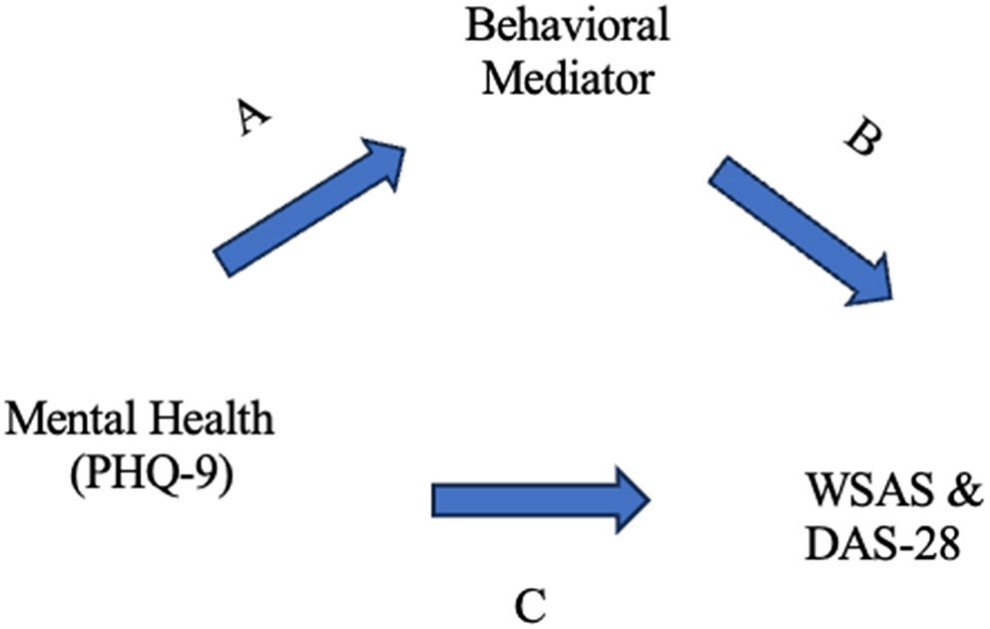
Mediation model

Finally, mediations between the mental health and disease outcome variables were completed using the behavioral factors of diet, physical activity, medication adherence, body mass index, insomnia alcohol intake, and smoking. The A path measured the association between mental health (depression or anxiety) and the behavioral mediator. The association between the behavioral mediator and the WSAS or DAS-28 was calculated in the B path, which was fixed to correspond to the 3-month outcomes. The analysis used a structural equation modelling approach with the (indirect) mediated effect estimated by the product of the coefficients (AxB) for the regression of the behavioral factor on depression and the disease outcome on the behavioral factor [38]. The proportion mediated was also calculated for the AxB paths. The direct effect C paths between mental health and the WSAS and DAS-28 outcomes were calculated. Lastly, the total effects paths (direct plus indirect paths) were also included. Variables included as potential confounders were age, gender, education, and baseline disease activity. Figure 1 displays the mediation model. All statistical analyses were completed in STATA 17.0.

## Results

### Sample characteristics

**Fig. 2:**
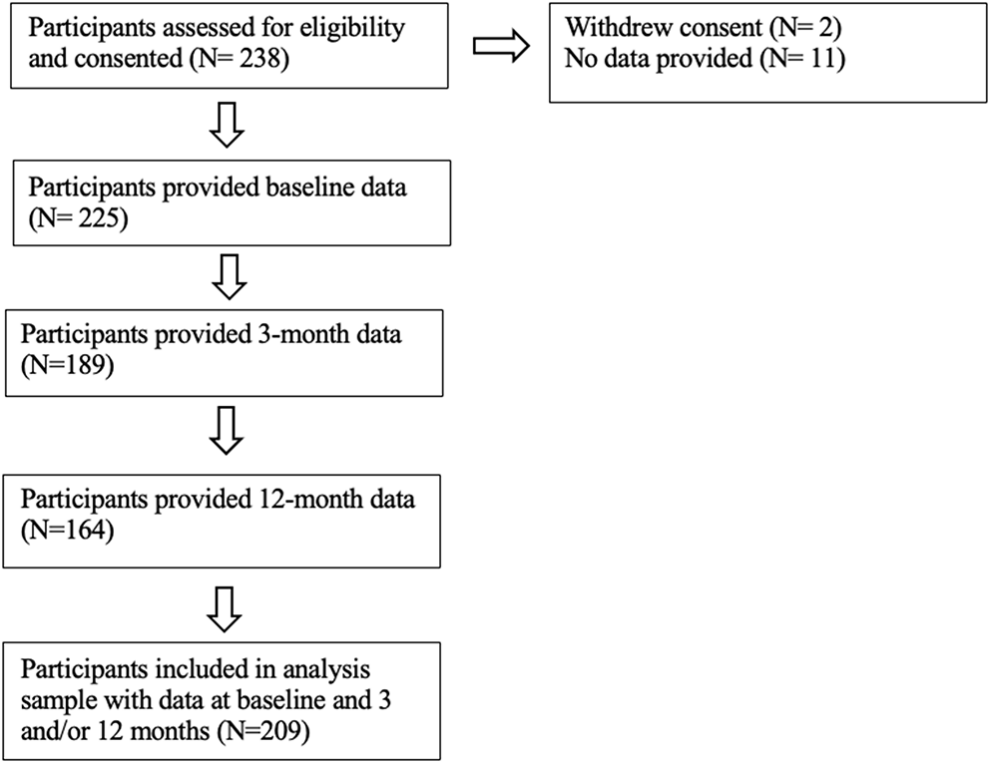
Flow chart of participation

**Fig. 3:**
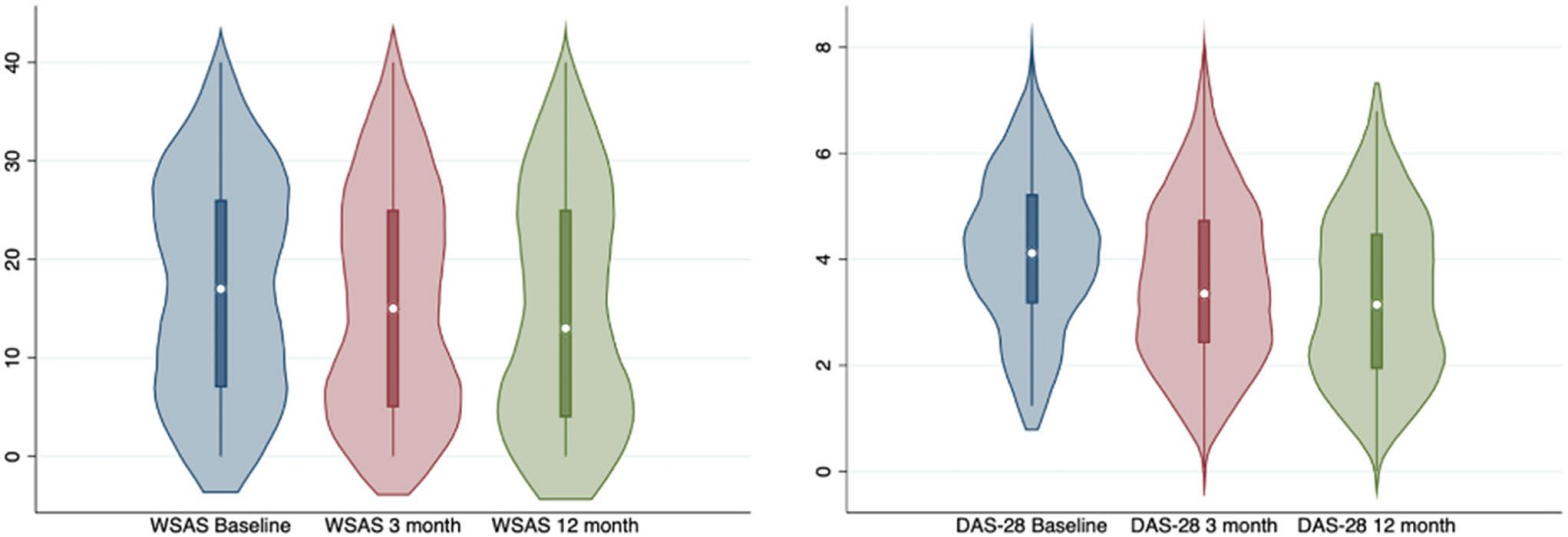
Violin plots of mean WSAS and DAS-28 scores over time

There were 209 participants included in the study. Figure 2 displays the flow chart of participation at each follow up, detailing the data collected at each time point. Table 1 displays the characteristics of the sample at baseline in terms of demographics and key variables. The overall sample had an average age of 53.2 years, was 72.4% female, and 66.7% white. Figure 3 displays the violin plots of the means for the WSAS and DAS-28 at baseline, 3 months, and 12 months. Given the differing rates of return at different timepoints and for patient versus clinical data, samples were compared in Table S1 in the Supplementary Material. There were no significant differences found between the samples with and without longitudinal data.

**Table 1:** Demographics table

	Total Sample Baseline
N	209
Age, Mean (SD)	53.2 (14.0)
Gender, %	72.4
Education, %	
None GCSE A-level Undergraduate Postgraduate Unknown	16.4 20.9 10.2 12.4 16.0 24.0
Ethnicity, %	
White Black Asian Multiracial Other Unknown	66.7 3.6 2.7 2.2 1.8 23.1
Depression (PHQ), Mean (SD)	13.9 (11.0)
Anxiety (GAD), Mean (SD)	5.4 (5.5)
Diet, Mean (SD)	7.55 (3.8)
Physical Activity (IPAQ), Mean (SD)	934.5 (1663.9)
Body Mass Index (BMI)	28.8 (6.6)
Alcohol (AUDIT), Mean (SD)	2.2 (2.3)
Insomnia (ISI), Mean (SD)	13.1 (5.3)
Disease Activity (DAS-28), Mean (SD)	4.2 (1.5)
Function (WSAS), Mean (SD)	17.3 (11.1)
Pain VAS, Mean (SD)	49.4 (25.3)
Fatigue VAS, Mean (SD)	57.1 (24.5)

GCSE: General Certificate of Secondary Education, PHQ: Patient Health Questionnaire, GAD: Generalized Anxiety Disorder, IPAQ: International Physical Activity Questionnaire, BMI: Body Mass Index, AUDIT: Alcohol Use Disorders Identification Test, ISI: Insomnia Severity Index, DAS−28: Disease Activity Score, WSAS: Work and Social Adjustment Scale, VAS: Visual Analog Scale

### Changes in outcomes over time

Violin plots (Fig. 3) were created showing the changes in mean scores of the WSAS and DAS-28 over time at baseline, 3 months, and 12 months. For both WSAS and DAS-28, the means decreased over time. The distribution of mean scores for the WSAS stayed relatively steady over time whereas the DAS-28 distribution became less clustered around the mean for the DAS-28 after the baseline. This develops into a less normal distribution over time with scores tending towards a bimodal distribution by 12 months for the DAS-28 whereas the WSAS starts with a bimodal shape then grows further in that direction. These distributions indicate that participants increasingly were clustering at low or high scores with fewer people scoring moderately over time. However, the low scores appear slightly more common than higher scores.

### Correlations between variables of interest at baseline and outcomes over time

Correlations between variables are presented in Supplementary Table S1. Key variables in the regression analyses were included in the matrix. Depression and anxiety correlations with the DAS-28 and WSAS remained relatively stable across baseline, 3 months, and 12 months, with both outcomes showing low to moderate correlations. All correlations between depression and anxiety indicated that worse mental health was associated with worse WSAS and DAS-28 scores.

For the behavioral variables, physical activity showed correlations in the expected directions with mental health and most of the behavioral factors except for alcohol. It showed mixed results with the disease outcomes over time. Diet showed mixed results across the correlations, appearing in the expected direction for some outcomes but the unexpected outcome for others. However, the correlations tended to be small either way. BMI showed correlations in the expected directions except for alcohol. The correlations for BMI were weak across all variables. Alcohol was highly mixed in that the correlations showed the expected direction for diet smoking, and insomnia as well as the disease outcomes, but mental health and the remaining behavioral factors showed correlations in the unexpected direction. Smoking showed correlations in the expected direction for all the behavioral variables except for medication adherence, though that correlation was very small. It also showed correlations in the expected direction for the disease outcomes and mental health variables. Lastly, medication adherence was mixed in that it showed correlations in the unexpected direction for the mental health variables, diet, alcohol, and smoking, but they were small. However, it was highly mixed for the correlations with behavioral variables.

### EULAR response

At 3 months, 12.4% had a moderate EULAR DAS-28 response, and 8% had a EULAR DAS-28 good response (Table 2). At 12 months, 9.8% had a moderate EULAR DAS-28 response, and 13.3% had a good EULAR DAS-28 response. The adjusted odds ratios for the EULAR DAS-28 response are shown in Table 3. These were adjusted for age, gender, and baseline DAS-28. Greater depression, both numerical and categorical, was not significantly associated with worsened EULAR response at any of the timepoints.

While the effects were in the anticipated direction, these results do not confirm a reduced chance of achieving EULAR remission with greater depression or anxiety scores. This was true for when they were used both categorically and as a numerical total score. However, although none of them were significant, the odds ratios did show a lower odds ratio for 3 and 12 months for both numerical and categorical depression and 3-month numerical depression. Thus, while the odds ratios were in the expected direction, they were not found to be significant. The same trend occurred for anxiety with the exception of the 12-month GAD total which had an increased OR, but this was not significant.

**Table 2:** Adjusted odds ratios for EULAR response criteria for depression and anxiety

	Odds Ratio	*P*	95% CI
**PHQ Total score**			
3 Month EULAR DAS-28	0.96	0.36	[0.89, 1.04]
12 Month EULAR DAS-28	0.99	0.99	[0.93, 1.07]
**PHQ categories** **None/Mild vs. Moderate/Severe**			
3 Month EULAR DAS-28	0.64	0.34	[0.26, 1.58]
12 Month EULAR DAS-28	0.67	0.44	[0.28,1.72]
**GAD Total score**			
3 Month EULAR DAS-28	0.98	0.76	[0.86, 1.09]
12 Month EULAR DAS-28	1.04	0.53	[0.92, 1.15]
**GAD categories** **None/Mild vs. Moderate/Severe**			
3 Month EULAR DAS-28	0.99	0.99	[0.71, 1.29]
12 Month EULAR DAS-28	0.94	0.75	[0.61, 1.27]

PHQ: Patient Health Questionnaire, EULAR: European Alliance of Associations for Rheumatology, DAS−28: Disease Activity Score, GAD: Generalized Anxiety Disorder

### Mediation models

The relationships between mental health and outcomes were tested to determine if behavioral factors influenced the relationship. First, depression and anxiety were used as the initial mental health factor and the WSAS and DAS-28 were used as outcomes at 3 and 12 months. The direct paths, indirect paths, and proportion mediated for depression and anxiety are shown in supplementary Tables S3-S8. Further details of the relationship between the mental health factors and disease outcomes are in the supplementary section.

The relationship between mental health and disease outcomes was mostly insignificant for the DAS-28 and WSAS at 3 and 12 months, with the exception of depression on the DAS-28 at 3 months. The effects differed slightly depending on the confounder adjusted for. A summary table is shown below in Table 3 with the full data presented in Table S6.

**Table 3:** Summary of C total paths

	WSAS 3 Month	WSAS 12 Month	DAS-28 3 Month	DAS-28 12 Month
**Depression**	b = 0.07, *p* = 0.39, 95% CI=[-0.09, 0.22]	b = 0.03, *p* = 0.74 95% CI=[-0.18, 0.25]	b = 0.21, *p* = 0.04 95% CI=[0.01, 0.41]	b = 0.15, *p* = 0.20 95% CI=[-0.08, 0.36]
Anxiety	b = 0.13, *p* = 0.08 95% CI=[-0.02, 0.28]	b = 0.07, *p* = 0.51 95% CI=[-0.13, 0.27]	b = 0.17, *p* = 0.14 95% CI=[-0.06, 0.39]	b = 0.06, *p* = 0.65 95% CI=[-0.20, 0.32]

^WSAS: Work and Social Adjustment Scale, DAS−28: Disease Activity Score^

Next, the behavioral factors of inflammatory diet, physical activity, medication adherence, body mass index, insomnia, alcohol, and smoking were tested for their role as mediators, or whether they impact the relationship between mental health and outcomes. Further details discussing the direct, indirect, and additional mediation results are also included in the supplementary section. Although diet was significantly associated with anxiety (b=-0.31, *p* = 0.01, 95% CI=[-0.55, -0.08]), it was not found to be a mediator between mental health and any disease outcomes. Physical activity did not show any significant associations with mental health or disease outcomes nor was it found to be a mediator. Medication adherence similarly had no significant associations with mental health or disease outcomes nor was it found to be a mediator. BMI was significantly associated with depression (b = 0.26, *p* = 0.04, 95% CI=[0.01, 0.51]), but not anxiety. For the disease outcomes, BMI was significantly associated with the WSAS for depression (b = 0.15, *p* = 0.02, 95% CI=[0.02, 0.28]) and anxiety (b = 0.12, *p* = 0.04, 95% CI=[0.01, 0.24]). However, it was not found to be a significant mediator. Insomnia was associated with depression (b = 0.36, *p* < 0.01, 95% CI=[0.17, 0.56]) and anxiety (b = 0.34, *p* < 0.01, 95% CI=[0.12, 0.55]). It was also associated with the WSAS but not the DAS-28. This was true for both the models using depression (b = 0.22, *p* < 0.01, 95% CI=[0.08, 0.35]) and anxiety (b = 0.21, *p* < 0.01, 95% CI=[0.08, 0.35]). It was also a significant mediator for depression (b = 0.08, *p* = 0.03, 95% CI=[0.01, 0.15]) and anxiety (b = 0.07, *p* = 0.03, 95% CI=[0.01, 0.13]) but only for the WSAS, not the DAS-28. Although alcohol had no significant associations with mental health or disease outcomes, it was a significant mediator in the depression model (b = 0.21, *p* = 0.04, 95% CI=[0.01, 0.41]), but not in the anxiety model. Smoking was not associated with mental health, but it was significantly associated with the DAS-28 in the depression model (b=-0.11, *p* = 0.03, 95% CI=[-0.21, -0.01]). It was not a significant mediator.

## Discussion

The first aim of this study was to determine if depression and anxiety are related with the disease outcomes of DAS-28 and WSAS over time. Firstly, it appears from the correlations that mental health is clearly related with disease outcomes. However, longitudinally these relationships do not appear to remain consistently strong even if they stay in the expected direction. Secondly, the current study also found the results to be nuanced between depression versus anxiety and for the DAS-28 versus the WSAS. While a significant relationship emerged between depression and the DAS-28 at 3 months, it appears it is only a short-term relationship and fades by 12 months. Since the only relationship between anxiety and the WSAS also appeared at 3 months but faded over time by 12 months, the overall results seem to indicate that the relationship between mental health and disease outcomes is only short-term.

The findings in this study that certain self-management behaviors are associated with mental health aligns with existing research on the relationship between mental health and behavioral factors [39–41]. Though many interventional studies with RA patients indicate the benefits of physical activity and healthy diet in self-management of RA, the role of mental health in those relationships has had little research within the RA population [42–44]. Given the strong links between RA and mental health conditions and the international recommendations for self-management [45], it is important for these relationships to be understood within the context of RA.

The next aim of this PROsPER-RA study was to evaluate the relationship between mental health and self-management behaviors. Insomnia emerged as having the clearest relationship with mental health as it held this association for both depression and anxiety. BMI and diet also appeared to be related with mental health, though they were more nuanced in terms of whether they related with depression or anxiety, which may be due to the limited questions on diet. This indicates that although insomnia shows a strong relationship with mental health, the remaining behavioral factors appear to have a more complex relationship. The differences between symptoms of depression versus anxiety may create differences in behavioral responses.

The relationship between self-management behaviors and disease outcomes was also evaluated. Insomnia also emerged as the self-management behavior most strongly related with disease outcomes, indicating its importance for RA patients. The clearer relationship for BMI with the WSAS compared with the DAS-28 potentially points to the differences between broader subjective measures of RA outcomes compared with more objective disease activity measures. The differing results between them suggest that impacts of RA may be better captured by broader measures that more disease-specific measures can miss. The remaining mixed results point to the possibility that a larger sample size may be needed, especially since many results were insignificant but in the expected direction. However, the power analysis indicated that there was unlikely to be an issue of low power.

It is also important to note that the self-management behaviors were reported during the Coronavirus Disease (COVID-19) lockdowns which had prompted many people to make changes to their health behaviors, some in healthy directions and some in unhealthy directions [46, 47]. The occasionally unexpected directions of the results could be reflecting the sudden changes to behaviors which took place during the lockdowns. It could also be capturing changes to risk perception among RA patients, who may have had increased vigilance of their self-management behaviors. While this limits the conclusions from this study, it also points to the malleability of self-management behaviors, which is encouraging for the potential of self-management interventions.

Finally, a few of the behavioral factors were found to mediate between mental health and disease outcomes. Insomnia again showed the strongest relationship as a mediator, underscoring its role in various aspects of RA. Alcohol also appeared to weakly mediate the relationship between mental health and RA outcomes, despite its unexpected direction. This could be reflecting that the relationship between self-management behaviors, mental health, and RA outcomes is complex. It may be that people who have experienced worse RA symptoms are more likely to avoid alcohol to avoid exacerbating symptoms further or those who are having few symptoms may be more relaxed towards self-management behaviors. This indicates that these behaviors may have unique expressions in RA patients and require further population-specific studies.

There were a number of strengths and limitations for this study. The longitudinal design allowed the study to detect differences over time, which helps inform the design of future research. Also, the diversity of health behaviors included demonstrated the variance in relationships that mental health may have with self-management health behaviors. The sample size was a limitation, which was somewhat compensated for by a full-information maximum likelihood, allowing for all people with at least baseline and either the 3- or the 12-month follow-ups to be included. Additionally, the majority of the sample was white, which also limits the generalizability of the results. Also, the DAS-28 includes components such as VAS and Patient Global Assessment (PGA) which could be influenced by mental health but can be difficult to disentangle. Similarly, patient reported outcomes are important to holistically address RA outcomes, but have the disadvantage of greater subjectivity and bias. Furthermore, the selection criteria, while broad enough to recruit a large number of participants, had the downside of the potential for confounding factors to exist which could reduce the external validity of the results. Lastly, diet was a very limited measure so the conclusions derived from results pertaining to diet are thus very limited. Future research may further investigate behavioral theories to improve implementation of self-management behaviors which would improve RA outcomes [48, 49].

In conclusion, mental health plays an important role in influencing RA outcomes and self-management health behaviors. This study demonstrated the impact of both mental health and self-management behaviors across various aspects of RA. It also studied behaviors in a unique pandemic context, demonstrating the malleability of self-management behaviors. In summary, the findings should encourage clinicians to address mental health and self-management with patients to improve RA disease outcomes.

## Electronic Supplementary Material

Below is the link to the electronic supplementary material.

Supplementary Material 1

Supplementary Material 2
