# Supplementary material for: Self-management behaviors do not affect remission but mediate between mental health and disease outcomes in a longitudinal study of rheumatoid arthritis
Source: Rheumatol Int. 2025 Jan 17;45(2):31. doi: 10.1007/s00296-024-05761-8 (PMC11741988; doi:10.1007/s00296-024-05761-8)
Supplement: Supplementary file 1 — Supplementary Material 1 [file 296_2024_5761_MOESM1_ESM.docx]

**Supplementary Information**

Table S1. Correlation matrix

|  | 1 | 2 | 3 | 4 | 5 | 6 | 7 | 8 | 9 | 10 | 11 | 12 | 13 | 14 |
| --- | --- | --- | --- | --- | --- | --- | --- | --- | --- | --- | --- | --- | --- | --- |
| 1. Depression | -- |  |  |  |  |  |  |  |  |  |  |  |  |  |
| 2. Anxiety | 0.94 | -- |  |  |  |  |  |  |  |  |  |  |  |  |
| 3. Physical Activity | -0.19 | -0.21 | -- |  |  |  |  |  |  |  |  |  |  |  |
| 4. Diet | -0.28 | -0.25 | 0.16 | -- |  |  |  |  |  |  |  |  |  |  |
| 5. Alcohol | -0.13 | -0.11 | 0.09 | -0.04 | -- |  |  |  |  |  |  |  |  |  |
| 6. Smoking | 0.14 | 0.12 | -0.21 | -0.22 | 0.07 | -- |  |  |  |  |  |  |  |  |
| 7. Insomnia | 0.56 | 0.52 | -0.14 | -0.05 | -0.09 | 0.01 | -- |  |  |  |  |  |  |  |
| Medication Nonadherence | 0.10 | 0.06 | 0.04 | -0.11 | 0.05 | 0.00 | 0.14 | -- |  |  |  |  |  |  |
| 8. BMI | 0.17 | 0.13 | -0.03 | -0.20 | -0.06 | 0.04 | 0.01 | 0.05 |  |  |  |  |  |  |
| 9. WSAS Baseline | 0.49 | 0.43 | -0.30 | -0.00 | -0.29 | 0.17 | 0.50 | -0.01 | -0.05 | -- |  |  |  |  |
| 10. DAS28 Baseline | 0.26 | 0.18 | -0.05 | 0.05 | -0.22 | 0.14 | 0.21 | 0.07 | 0.09 | 0.43 | -- |  |  |  |
| 11. WSAS –  3 Month | 0.42 | 0.4 | -0.17 | -0.01 | -0.24 | 0.29 | 0.30 | 0.05 | 0.15 | 0.71 | 0.58 | -- |  |  |
| 13. DAS 28 - 3 Month | 0.31 | 0.26 | 0.02 | -0.21 | -0.18 | 0.01 | 0.18 | 0.08 | 0.08 | 0.17 | 0.52 | 0.42 | -- |  |
| 14. WSAS – 12 Month | 0.34 | 0.32 | 0.01 | 0.26 | -0.06 | 0.02 | 0.56 | 0.06 | 0.09 | 0.62 | 0.31 | 0.73 | 0.20 | -- |
| 15. DAS 28 – 12 Month | 0.20 | 0.10 | -0.03 | 0.02 | -0.04 | 0.12 | 0.31 | 0.10 | 0.04 | 0.24 | 0.53 | 0.35 | 0.48 | 0.46 |

Table S2. Baseline characteristics of the subsample with longitudinal data

|  | **Sample without Longitudinal Data** | **Sample with Longitudinal Data** | **P** |
| --- | --- | --- | --- |
| N | 16 | 209 |  |
| **Age, Mean (SD)** | 53.1 (13.5) | 53.2 (14.1) | 0.99 |
| **Gender, %** | 30% | 22.2% | 0.56 |
| **Education, %**  **None**  **GCSE**  **A-level**  **Undergraduate**  **Postgraduate**  **Unknown** | 25.0%  0.0%  12.5%  12.5%  6.2%  43.8% | 15.8%  22.5%  10.0%  12.4%  16.7%  22.5% | 0.14 |
| **Depression (PHQ), Mean (SD)** | 6.3 (6.1) | 8.6 (6.1) | 0.27 |
| **Anxiety (GAD), Mean (SD)** | 5.4 (7.2) | 5.4 (5.4) | 1.00 |
| **Diet, Mean (SD)** | 7.0 (4.7) | 7.6 (3.8) | 0.70 |
| **Physical Activity (IPAQ), Mean (SD)** | 442.0 (1050.7) | 957.4 (1686.3) | 0.50 |
| **Alcohol (AUDIT), Mean (SD)** | 2.4 (2.6) | 2.2 (2.3) | 0.80 |
| **Insomnia (ISI), Mean (SD)** | 9.3 (8.4) | 13.3 (5.1) | 0.05 |
| **Disease Activity (DAS-28), Mean (SD)** | 4.2 (1.3) | 4.2 (1.5) | 0.90 |
| **Function (WSAS), Mean (SD)** | 14.3 (11.5) | 17.4 (11.1) | 0.50 |
| **Pain VAS** | 42.8 (31.3) | 49.8 (24.9) | 0.40 |
| **Fatigue VAS** | 46.6 (30.1) | 57.7 (24.2) | 0.20 |

Table S3. A paths

| **Mediator** | **Depression as predictor** | | | **Anxiety as predictor** | | |
| --- | --- | --- | --- | --- | --- | --- |
|  | **A** | **P** | **95% CI** | **A** | **P** | **95% CI** |
| **Diet** | -0.16 | 0.19 | [-0.40, 0.08] | -0.31 | **0.01** | [-0.55, -0.08] |
| **Physical Activity** | -0.12 | 0.39 | [-0.41, 0.17] | -0.12 | 0.25 | [-0.34, 0.09] |
| **Medication**  **Nonadherence** | 0.16 | 0.15 | [-0.06, 0.37] | 0.07 | 0.49 | [-0.13, 0.27] |
| **Body Mass Index** | 0.26 | **0.04** | [0.01, 0.51] | 0.11 | 0.33 | [-0.11, 0.32] |
| **Insomnia** | 0.36 | **<0.01** | [0.17, 0.56] | 0.34 | **<0.01** | [0.12, 0.55] |
| **Alcohol** | -0.00 | 0.97 | [-0.24, 0.23] | -0.01 | 0.89 | [-0.20, 0.17] |
| **Smoking** | 0.11 | 0.29 | [-0.09, 0.32] | 0.09 | 0.40 | [-0.12, 0.29] |

Table S4. B paths

| **Mediator** | **Outcome** | **Depression as predictor** | | | **Anxiety as predictor** | | |
| --- | --- | --- | --- | --- | --- | --- | --- |
|  |  | **B** | **P** | **95% CI** | **B** | **P** | **95% CI** |
| **Diet** | WSAS | 0.07 | 0.28 | [-0.06, 0.21] | 0.11 | 0.13 | [-0.03, 0.24] |
|  | DAS-28 | 0.11 | 0.26 | [-0.08, 0.32] | 0.11 | 0.34 | [-0.11, 0.34] |
| **Physical**  **Activity** | WSAS | -0.08 | 0.08 | [-0.18, 0.01] | -0.08 | 0.08 | [-0.18, 0.01] |
|  | DAS-28 | 0.09 | 0.33 | [-0.09, 0.27] | 0.10 | 0.30 | [-0.09, 0.29] |
| **Medication**  **Nonadherence** | WSAS | 0.05 | 0.54 | [-0.11, 0.21] | 0.05 | 0.49 | [-0.09, 0.20] |
|  | DAS-28 | 0.06 | 0.39 | [-0.07, 0.18] | 0.07 | 0.27 | [-0.06, 0.21] |
| **Body Mass**  **Index** | WSAS | 0.15 | **0.02** | [0.02, 0.28] | 0.12 | **0.04** | [0.01, 0.24] |
|  | DAS-28 | 0.22 | 0.06 | [-0.01, 0.45] | 0.24 | **0.04** | [0.01, 0.46] |
| **Insomnia** | WSAS | 0.22 | **<0.01** | [0.08, 0.35] | 0.21 | **<0.01** | [0.08, 0.35] |
|  | DAS-28 | 0.00 | 0.98 | [-0.19, 0.20] | 0.01 | 0.94 | [-0.21, 0.22] |
| **Alcohol** | WSAS | -0.02 | 0.79 | [-0.16, 0.12] | -0.02 | 0.79 | [-0.16, 0.12] |
|  | DAS-28 | -0.06 | 0.44 | [-0.21, 0.09] | -0.06 | 0.45 | [-0.22, 0.09] |
| **Smoking** | WSAS | 0.05 | 0.32 | [-0.05, 0.15] | 0.05 | 0.33 | [-0.5, 0.15] |
|  | DAS-28 | -0.11 | **0.03** | [-0.21, -0.01] | -0.10 | 0.08 | [-0.21, 0.01] |

Table S5. C direct path

| **Mediator** | **Outcome** | **Depression as predictor** | | | **Anxiety as predictor** | | |
| --- | --- | --- | --- | --- | --- | --- | --- |
|  |  | **C Direct** | **P** | **95% CI** | **C Direct** | **P** | **95% CI** |
| **Diet** | WSAS 3 Month | 0.08 | 0.34 | [-0.08, 0.24] | 0.16 | **0.04** | [0.01, 0.32] |
|  | WSAS 12 Month | 0.05 | 0.67 | [-0.17, 0.23] | 0.10 | 0.34 | [-0.10, 0.31] |
|  | DAS-28 3 Month | 0.23 | **0.03** | [0.02, 0.44] | 0.20 | 0.10 | [-0.04, 0.45] |
|  | DAS-28 12 Month | 0.15 | 0.19 | [-0.06, 0.39] | 0.10 | 0.49 | [-0.18, 0.37] |
| **Physical Activity** | WSAS 3 Month | 0.05 | 0.53 | [-0.11, 0.20] | 0.12 | 0.13 | [-0.03, 0.26] |
|  | WSAS 12 Month | -0.02 | 0.87 | [-0.23, 0.19] | 0.01 | 0.91 | [-0.19, 0.21] |
|  | DAS-28 3 Month | 0.20 | **0.05** | [-0.09, 0.34] | 0.18 | 0.13 | [-0.05, 0.40] |
|  | DAS-28 12 Month | 0.12 | 0.30 | [-0.09, 0.34] | 0.04 | 0.76 | [-0.22, 0.30] |
| **Medication**  **Nonadherence** | WSAS 3 Month | 0.05 | 0.56 | [-0.11, 0.21] | 0.12 | 0.11 | [-0.03, 0.28] |
|  | WSAS 12 Month | -0.01 | 0.89 | [-0.23, 0.19] | 0.02 | 0.81 | [-0.17, 0.22] |
|  | DAS-28 3 Month | 0.20 | **0.05** | [-0.00, 0.40] | 0.17 | 0.13 | [-0.05, 0.39] |
|  | DAS-28 12 Month | 0.13 | 0.27 | [-0.09, 0.35] | 0.05 | 0.69 | [-0.21, 0.32] |
| **Body Mass Index** | WSAS 3 Month | 0.02 | 0.76 | [-0.13, 0.18] | 0.10 | 0.18 | [-0.05, 0.26] |
|  | WSAS 12 Month | -0.03 | 0.82 | [-0.25, 0.19] | 0.00 | 0.97 | [-0.20, 0.21] |
|  | DAS-28 3 Month | 0.16 | 0.15 | [-0.06, 0.37] | 0.14 | 0.17 | [-0.06, 0.35] |
|  | DAS-28 12 Month | 0.08 | 0.48 | [-0.14, 0.30] | 0.02 | 0.86 | [-0.24, 0.28] |
| **Insomnia** | WSAS 3 Month | -0.01 | 0.89 | [-0.17, 0.15] | 0.05 | 0.51 | [-0.10, 0.20] |
|  | WSAS 12 Month | -0.06 | 0.54 | [-0.26, 0.14] | -0.04 | 0.66 | [-0.23, 0.15] |
|  | DAS-28 3 Month | 0.20 | **0.05** | [<0.01, 0.41] | 0.17 | 0.16 | [-0.06, 0.41] |
|  | DAS-28 12 Month | 0.13 | 0.25 | [-0.09, 0.35] | 0.04 | 0.76 | [-0.24, 0.33] |
| **Alcohol** | WSAS 3 Month | 0.06 | 0.44 | [-0.09, 0.22] | 0.13 | 0.10 | [-0.02, 0.28] |
|  | WSAS 12 Month | 0.01 | 0.95 | [-0.21, 0.22] | 0.03 | 0.79 | [-0.18, 0.23] |
|  | DAS-28 3 Month | 0.21 | **0.04** | [0.01, 0.41] | 0.17 | 0.12 | [-0.05, 0.39] |
|  | DAS-28 12 Month | 0.13 | 0.24 | [-0.09, 0.36] | 0.04 | 0.73 | [-0.22, 0.31] |
| **Smoking** | WSAS 3 Month | 0.05 | 0.55 | [-0.11, 0.22] | 0.12 | 0.13 | [-0.03, 0.27] |
|  | WSAS 12 Month | 0.00 | 0.97 | [-0.21, 0.23] | 0.01 | 0.88 | [-0.19, 0.22] |
|  | DAS-28 3 Month | 0.22 | **0.03** | [0.02, 0.42] | 0.17 | 0.12 | [-0.04, 0.40] |
|  | DAS-28 12 Month | 0.13 | 0.26 | [-0.09, 0.35] | 0.04 | 0.76 | [-0.22, 0.31] |

Table S6. C total path

| **Mediator** | **Outcome** | **Depression as predictor** | | | **Anxiety as predictor** | | |
| --- | --- | --- | --- | --- | --- | --- | --- |
|  |  | **C Total** | **P** | **95% CI** | **C Total** | **P** | **95% CI** |
| **Diet** | WSAS 3 Month | 0.07 | 0.39 | [-0.09, 0.22] | 0.13 | 0.08 | [-0.02, 0.28] |
|  | WSAS 12 Month | 0.03 | 0.74 | [-0.18, 0.25] | 0.07 | 0.51 | [-0.13, 0.27] |
|  | DAS-28 3 Month | 0.21 | **0.04** | [0.01, 0.41] | 0.17 | 0.14 | [-0.06, 0.39] |
|  | DAS-28 12 Month | 0.15 | 0.20 | [-0.08, 0.36] | 0.06 | 0.65 | [-0.20, 0.32] |
| **Physical Activity** | WSAS 3 Month | 0.06 | 0.75 | [-0.10, 0.22] | 0.12 | 0.10 | [-0.02, 0.28] |
|  | WSAS 12 Month | -0.01 | 0.95 | [-0.22, 0.20] | 0.02 | 0.83 | [-0.18, 0.22] |
|  | DAS-28 3 Month | 0.19 | 0.06 | [-0.00, 0.40] | 0.16 | 0.15 | [-0.06, 0.38] |
|  | DAS-28 12 Month | 0.11 | 0.32 | [-0.11, 0.34] | 0.03 | 0.83 | [[-0.24, 0.29] |
| **Medication**  **Nonadherence** | WSAS 3 Month | 0.06 | 0.49 | [-0.11, 0.21] | 0.13 | 0.10 | [-0.02, 0.28] |
|  | WSAS 12 Month | -0.01 | 0.94 | [-0.22, 0.20] | 0.03 | 0.78 | [-0.17, 0.23] |
|  | DAS-28 3 Month | 0.21 | **0.04** | [0.01, 0.41] | 0.17 | 0.12 | [-0.04, 0.39] |
|  | DAS-28 12 Month | 0.13 | 0.23 | [-0.09, 0.35] | 0.06 | 0.67 | [-0.21, 0.32] |
| **Body Mass Index** | WSAS 3 Month | 0.06 | 0.44 | [0.09, 0.22] | 0.12 | 0.13 | [-0.04, 0.27] |
|  | WSAS 12 Month | 0.01 | 0.93 | [-0.21, 0.23] | 0.01 | 0.88 | [-0.19, 0.21] |
|  | DAS-28 3 Month | 0.21 | **0.03** | [0.01, 0.40] | 0.17 | 0.12 | [-0.04, 0.38] |
|  | DAS-28 12 Month | 0.14 | 0.22 | [-0.08, 0.35] | 0.05 | 0.71 | [-0.21, 0.31] |
| **Insomnia** | WSAS 3 Month | 0.07 | 0.39 | [-0.09, 0.22] | 0.12 | 0.11 | [-0.29, 0.27] |
|  | WSAS 12 Month | 0.01 | 0.89 | [-0.19, 0.22] | 0.02 | 0.79 | [-0.17, 0.22] |
|  | DAS-28 3 Month | 0.21 | **0.04** | [0.01, 0.42] | 0.17 | 0.12 | [-0.05, 0.39] |
|  | DAS-28 12 Month | 0.13 | 0.24 | [-0.09, 0.35] | 0.05 | 0.73 | [-0.22, 0.31] |
| **Alcohol** | WSAS 3 Month | 0.06 | 0.78 | [-0.09, 0.22] | 0.13 | 0.10 | [-0.02, 0.28] |
|  | WSAS 12 Month | 0.01 | 0.95 | [-0.21, 0.22] | 0.03 | 0.79 | [-0.18, 0.23] |
|  | DAS-28 3 Month | 0.21 | **0.04** | [0.01, 0.41] | 0.17 | 0.12 | [-0.05, 0.39] |
|  | DAS-28 12 Month | 0.13 | 0.24 | [-0.09, 0.36] | 0.05 | 0.73 | [-0.22, 0.31] |
| **Smoking** | WSAS 3 Month | 0.06 | 0.50 | [-0.11, 0.22] | 0.12 | 0.11 | [-0.03, 0.28] |
|  | WSAS 12 Month | 0.01 | 0.93 | [-0.21, 0.23] | 0.02 | 0.85 | [-0.18, 0.22] |
|  | DAS-28 3 Month | 0.21 | **0.04** | [0.01, 0.41] | 0.17 | 0.14 | [-0.06, 0.39] |
|  | DAS-28 12 Month | 0.13 | 0.26 | [-0.09, 0.35] | 0.03 | 0.81 | [-0.24, 0.30] |

Table S7. AxB mediations paths

| **Mediator** | **Outcome** | **Depression as predictor** | | | **Anxiety as predictor** | | |
| --- | --- | --- | --- | --- | --- | --- | --- |
|  |  | **AxB** | **P** | **95% CI** | **AxB** | **P** | **95% CI** |
| **Diet** | WSAS | -0.01 | 0.38 | [-0.04, 0.01] | -0.03 | 0.19 | [-0.08, 0.02] |
|  | DAS-28 | -0.02 | 0.34 | [-0.05, 0.02] | -0.03 | 0.32 | [-0.11, 0.03] |
| **Physical Activity** | WSAS | 0.01 | 0.49 | [-0.02, 0.04] | 0.01 | 0.38 | [-0.01, 0.03] |
|  | DAS-28 | -0.01 | 0.52 | [-0.04, 0.02] | -0.01 | 0.43 | [-0.04, 0.02] |
| **Medication**  **Nonadherence** | WSAS | 0.01 | 0.55 | [-0.02, 0.03] | 0.00 | 0.66 | [-0.01, 0.02] |
|  | DAS-28 | 0.01 | 0.47 | [-0.01,0.03] | 0.00 | 0.55 | [-0.01, 0.02] |
| **Body Mass Index** | WSAS | 0.04 | 0.14 | [-0.01, 0.08] | 0.01 | 0.39 | [-0.02, 0.04] |
|  | DAS-28 | 0.06 | 0.19 | [-0.03, 0.14] | 0.02 | 0.41 | [-0.03, 0.08] |
| **Insomnia** | WSAS | 0.08 | **0.03** | [0.01, 0.15] | 0.07 | **0.03** | [0.01, 0.13] |
|  | DAS-28 | 0.00 | 0.98 | [-0.07, 0.07] | 0.00 | 0.94 | [-0.07, 0.07] |
| **Alcohol** | WSAS | 0.00 | 0.98 | [-0.00, 0.00] | 0.00 | 0.91 | [-0.00, 0.00] |
|  | DAS-28 | 0.21 | **0.04** | [0.01, 0.41] | 0.00 | 0.89 | [-0.01, 0.01] |
| **Smoking** | WSAS | 0.01 | 0.54 | [-0.01, 0.02] | 0.00 | 0.54 | [-0.01, 0.02] |
|  | DAS-28 | -0.01 | 0.26 | [-0.03, 0.01] | -0.01 | 0.43 | [-0.03, 0.01] |

Table S8. Proportion mediated

| **Mediator** | **Outcome** | **Depression as predictor** | | | **Anxiety as predictor** | | |
| --- | --- | --- | --- | --- | --- | --- | --- |
|  |  | **Proportion**  **mediated** | **P** | **95% CI** | **Proportion**  **mediated** | **P** | **95% CI** |
| **Diet** | WSAS 3 Month | -0.18 | 0.49 | [-0.69, 0.33] | -0.25 | 0.29 | [-0.73, 0.22] |
|  | WSAS 12 Month | -0.33 | 0.75 | [-2.35, 1.70] | -0.48 | 0.56 | [-2.09, 1.12] |
|  | DAS-28 3 Month | -0.33 | 0.75 | [-2.35, 1.70] | -0.48 | 0.56 | [-2.09, 1.13] |
|  | DAS-28 12 Month | -0.09 | 0.37 | [-0.28, 0.10] | -0.21 | 0.37 | [-0.67, 0.25] |
| **Physical Activity** | WSAS 3 Month | -0.13 | 0.44 | [-0.45, 0.19] | -0.57 | 0.68 | [-3.26, 2.11] |
|  | WSAS 12 Month | 0.18 | 0.58 | [-0.46, 0.81] | 0.08 | 0.41 | [-0.12, 0.28] |
|  | DAS-28 3 Month | -1.42 | 0.95 | [-42.82, 39.98] | 0.47 | 0.84 | [-4.10, 5.04] |
|  | DAS-28 12 Month | -0.06 | 0.53 | [-0.23, 0.12] | -0.08 | 0.46 | [-0.28, 0.13] |
| **Medication**  **Nonadherence** | WSAS 3 Month | -0.10 | 0.62 | [-0.49, 0.29] | -0.45 | 0.84 | [-4.86, 3.97] |
|  | WSAS 12 Month | 0.14 | 0.65 | [-0.46, 0.74] | 0.03 | 0.68 | [-0.11, 0.16] |
|  | DAS-28 3 Month | 0.92 | 0.94 | [-25.15, 23.31] | 0.12 | 0.82 | [-0.93, 1.17] |
|  | DAS-28 12 Month | 0.04 | 0.51 | [-0.08, 0.16] | 0.03 | 0.57 | [-0.07, 0.13] |
| **Body Mass Index** | WSAS 3 Month | 0.06 | 0.63 | [-0.13, 0.26] | 0.09 | 0.72 | [-0.40, 0.57] |
|  | WSAS 12 Month | 0.60 | 0.46 | [-0.99, 2.20] | 0.11 | 0.43 | [-0.16, 0.39] |
|  | DAS-28 3 Month | 3.84 | 0.93 | [-86.78, 94.45] | 0.78 | 0.88 | [-9.52, 11.09] |
|  | DAS-28 12 Month | 0.27 | 0.26 | [-0.20, 0.74] | 0.15 | 0.42 | [-0.21, 0.51] |
| **Insomnia** | WSAS 3 Month | 0.42 | 0.33 | [-0.42, 1.23] | 0.51 | 0.72 | [-2.28, 3.29] |
|  | WSAS 12 Month | 1.15 | 0.40 | [-1.56, 3.88] | 0.58 | 0.15 | [-0.21, 1.38] |
|  | DAS-28 3 Month | 5.59 | 0.89 | [-78.27, 89.45] | 2.67 | 0.79 | [-17.21, 22.56] |
|  | DAS-28 12 Month | 0.00 | 0.98 | [-0.33, 0.35] | 0.02 | 0.94 | [-0.41, 0.45] |
| **Alcohol** | WSAS 3 Month | 0.01 | 0.98 | [-0.54, 0.55] | 0.06 | 0.94 | [-1.57, 1.68] |
|  | WSAS 12 Month | 0.00 | 0.98 | [-0.07, 0.07] | 0.00 | 0.91 | [-0.03, 0.03] |
|  | DAS-28 3 Month | 0.01 | 0.98 | [-0.65, 0.67] | 0.01 | 0.92 | [-0.16, 0.18] |
|  | DAS-28 12 Month | 0.00 | 0.98 | [-0.06, 0.07] | 0.00 | 0.89 | [-0.06, 0.07] |
| **Smoking** | WSAS 3 Month | 0.00 | 0.98 | [-0.10, 0.10] | 0.01 | 0.90 | [-0.24, 0.27] |
|  | WSAS 12 Month | 0.10 | 0.66 | [-0.35, 0.56] | 0.03 | 0.56 | [-0.08, 0.15] |
|  | DAS-28 3 Month | 0.55 | 0.93 | [-11.89, 12.99] | 0.21 | 0.86 | [-2.06, 2.47] |
|  | DAS-28 12 Month | -0.06 | 0.34 | [-0.18, 0.06] | -0.05 | 0.52 | [-0.21, 0.11] |

*Mental health and disease outcomes*

The relationship between mental health and disease outcomes at 3 and 12 months was tested. The C total path (Table S5) controls for potential confounders only, whereas the C direct path (Table S4) is both confounder and mediator adjusted. As the C total paths vary only slightly between models with mediators, since they are not adjusted for mediators, we report only the effects for the model including diet.

The total effect of depression on DAS-28 at 3 months is significant (b=0.21, p=0.04) but the results are insignificant for the WSAS at 3 months (b=0.07, p=0.39) and 12 months (b=0.03, p=0.74) and the DAS-28 at 12 months (b=0.15, p=0.20). The C total paths for anxiety are insignificant for all outcomes: WSAS at 3 months (b=0.13, p=0.08), WSAS at 12 months (b=0.07, p-0.51), DAS-28 3 months (b=0.17, p=0.14), and the DAS-28 at 12 months (b=0.06, p=0.65). Compared with the C direct path, the results are only slightly different. The C direct paths for diet are only significant for depression with the DAS-28 at 3 months again (b=0.23, p=0.03) while the remaining results are insignificant for the WSAS at 3 months (b=0.08, p=0.34), WSAS at 12 months (b=0.05, p=0.67), and the DAS-28 at 12 months (b=0.15, p=0.19). The results for anxiety are significant for the WSAS at 3 months but have a small effect (b=0.16, p=0.04) while the remaining results are insignificant for the WSAS at 12 months (b=0.10, p=0.34), DAS-28 at 3 months (b=0.20, p=0.10), and the DAS-28 at 12 months (b=0.10, p=0.49). These comparisons between the C direct and C total paths show that including the mediating variable can slightly alter the results, but any mediation effects were small so the C direct path results did not vary much compared with the C total path results.

The results for depression or anxiety and the WSAS or DAS-28 are displayed in table 8.6. Depression was significantly associated with the DAS-28 at 3 months (b=0.23, p=0.03), but was not significantly associated with any other outcome of the WSAS or DAS-28. The results varied slightly depending which mediator was used in the model in the sense that controlling for them changed the effects sizes slightly, but all showed the same pattern of significance only for the DAS-28 at 3 months. When considering all of the mediation models, some had effects in the unexpected direction, but they were very small and insignificant while nearly all effects were in the expected direction, even if they were insignificant. These findings show that the DAS-28 at 3 months appeared to have the strongest relationship with depression. Comparing these findings with the correlation results indicates that while there were moderately sized correlations between depression and the WSAS and DAS-28 outcomes at all timepoints, only the DAS-28 at 3 months was significant when the associations were tested in the mediation models, which included controlling for confounding variables. Thus, the relationship between depression and disease appears to be short-term and only present for disease activity but not quality of life, though these conclusions would be strengthened from replication in additional cohorts.

Anxiety was significantly associated with the WSAS at 3 months (b=0.16, p=0.04), but only in the mediation model for diet. The effect sizes of the remaining models were slightly smaller than the effect size in the diet model so they did not reach statistical significance. These results indicate that controlling for behavioral variables slightly changes the relationship between anxiety and disease outcomes. However, regardless of which behavioral mediator was included in the model, the relationship between anxiety and the WSAS or DAS-28 were all in the expected direction, with the exception of the WSAS at 12 months in the insomnia model, but this effect was small and insignificant. Overall, it appears that the relationship between anxiety and disease outcomes is weaker than for depression and varies when behavioral variables are controlled for. Comparing these results with the correlations, the correlations with anxiety and the disease outcomes were slightly smaller than for depression except for the WSAS at 3 months. This aligns with these results from the associations in the mediation model which show a weaker relationship for anxiety and disease outcomes compared with depression.

Next, the indirect effects were calculated for each of the mediating behavioral factors. Tables S2 and S3 show the A and B paths of the mediation models respectively for each of the behavioral factors. The A path tests the direct effect of the mental health predictor on the outcome of WSAS or DAS-28 while the B path tests the effect of the behavioral mediator on the outcome. The overall indirect AxB paths are displayed in table S6. The proportion mediated for the indirect paths are displayed in table S6. The total effects are included in table S5.

*Diet*

Diet did not show a significant association with depression when the A path was tested. However, it was in the expected direction, indicating healthier diet was corresponded with less depression. Anxiety, on the other hand, was found to be significantly associated with diet (b=-0.31, p=0.01) and in the expected direction such that better diet was linked with lower anxiety.

The relationship between diet and the WSAS and DAS-28 was then tested in the B paths. The effects were insignificant but in the unexpected direction, indicating worse diet corresponded to better WSAS or DAS-28 scores. However, these effects were small, insignificant, and their 95% confidence interval included negative effects which indicate that the expected direction of the effect was included in the range. However, to further investigate this unexpected direction in the B paths, it was tested if the model was overcontrolling for variables. The comorbidities variable was thus removed as the only non-demographic variable which had been controlled for. While this slightly reduced the effect size in the WSAS for anxiety (b=0.07, p=0.29), it did not reduce the effect size for the DAS-28 (b=0.11, p=0.32). The same was true for depression in that the WSAS was slightly reduced at 3 months (b=0.05, p=0.43), but the effect size remained the same for the DAS-28 (b=0.11, p=0.23) when comorbidities were not controlled for. The association using baseline diet scores rather than 3-month scores was also tested to determine if the unexpected direction was related with time differences. In the depression model, this did not alter the results for the WSAS in that they were still in the unexpected direction (b=0.09, p=0.14). However, for the DAS-28, the effect was in the expected direction, though still insignificant (b=-0.03, p=0.69). In the anxiety model, the effect also remained similarly in the unexpected direction for the WSAS (b=0.08, p=0.15), but flipped to the expected direction for the DAS-28 (b=-0.06, p=0.48). Though the effects were still insignificant and very small. This indicates that the relationship between diet and WSAS or DAS-28 may be dependent on time, with perhaps a lag in its effect. The lagged effect appeared to only be true for the DAS-28 so may be more related with more direct disease activity rather than quality of life.

The overall mediating path was also tested to determine whether diet mediated between mental health and disease outcomes. This AxB path was insignificant for diet in both the depression and anxiety models. This was true for both the WSAS and DAS-28 outcomes. The corresponding proportion mediated paths, which indicate how much of the effect occurs through the mediating variable, was also insignificant as would be expected when the mediating paths were insignificant. Thus, while diet shows some significant associations with mental health and disease outcomes, it does not appear to significantly mediate the relationship.

*Physical activity*

Physical activity was tested for the A path to determine if there was a significant association between mental health and physical activity. It did not show any significant association for either depression or anxiety. However, the effects were in the expected direction, indicating that more physical activity corresponded with lower depression and anxiety.

In the B path, which was testing the relationship between physical activity and the disease outcomes of WSAS or DAS-28, there were no significant associations found. This was true in both the depression and anxiety models, indicating that controlling for those variables did not change the results. Although they were not significant, the effects were in the expected direction for the WSAS for both depression and anxiety models. For the DAS-28, the effects were in the unexpected direction, but they were small, insignificant, and included the expected direction in their 95% confidence intervals, indicating minimal influence. Comparing these results with the correlations, the results similarly indicate a weak relationship between physical activity and all of the disease outcomes at all timepoints.

Finally, the overall mediating path was tested to determine if physical activity mediated between mental health and disease outcomes. The AxB path was insignificant for both the depression and anxiety models and for both the WSAS and DAS-28. The proportion mediated was also insignificant as expected. These results align with the findings of the A and B paths all being insignificant since it would follow that the overall mediating path would also be insignificant. Thus, physical activity does not appear to mediate between mental health and disease outcomes.

*Medication nonadherence*

Medication nonadherence was tested for the A path to determine if there was a significant association with mental health. It was found that medication adherence was not significantly associated with depression or anxiety. Compared with the correlations, the insignificant associations from the mediation model echo the small sizes of the correlations.

In the B path, which was testing the relationship between medication nonadherence and disease outcomes of the WSAS or DAS-28, there were no significant associations found. This was true in both the depression and anxiety models. Although they were not significant, the effects were in the expected direction for all of the models. These findings indicate that medication adherence does not appear to be significantly related with WSAS or DAS-28 outcomes. Comparing these results with the correlations, the insignificant associations appear to similarly indicate a weak relationship between medication adherence and WSAS or DAS-28 outcomes.

The overall mediating path was tested to determine if medication nonadherence mediated between mental health and disease outcomes. This AxB was insignificant as expected given the insignificant associations in the A and B paths between medication adherence and mental health or disease outcomes. It follows that the proportions mediated were also small and insignificant.

*BMI*

BMI was tested for the A path to determine if there was a significant association with mental health. There was a significant association with depression (b=0.24, p=0.04), but not with anxiety (b=0.11, p=0.33). However, BMI was in the expected direction with anxiety. These findings indicate that BMI is more strongly linked with depression than anxiety. Compared with the correlations, these results match the larger size correlation found with depression and BMI compared to anxiety.

In the B path, which tested the relationship between BMI and disease outcomes of the WSAS or DAS-28, BMI was significantly associated with the WSAS for both depression (b=0.15, p=0.02) and anxiety (b=0.12, p=0.04). However, with the DAS-28, BMI only showed a significant association with anxiety (b=0.21, p<0.01), but not depression. It was still in the expected direction though with depression even though it was not significant. These results suggest that BMI is clearly related with the WSAS when controlling for either depression or anxiety but is only related with the DAS-28 when controlling for anxiety. Compared with the correlations, the directions align with these results, but are a reminder that the relationship has a small effect even when significant.

The overall mediating path, AxB, was tested to determine if BMI mediated between mental health and disease outcomes. None of the mediation models were significant, indicating that despite some of the significant associations between BMI and mental health or disease outcomes, that BMI does not significantly mediate between mental health and disease outcomes. The proportion mediated results match these findings in that they are also insignificant.

*Insomnia*

Insomnia was tested in the A path to determine if there was a significant association with mental health. There was a significant association with insomnia in both the models with depression (b=0.36 p<0.01) and with anxiety (b=0.34, p<0.01). These results indicate that insomnia has a clear relationship with mental health. Comparing these results with the correlations, it appears that the correlations between insomnia and mental health were the largest of any of the behavioral variables, supporting the findings from the mediation models indicating a strong relationship.

In the B path, which tested the relationship between insomnia and disease outcomes of the WSAS or DAS-28, insomnia was significantly associated with the WSAS, but not the DAS-28. The association with the WSAS was significant in both the models using depression (b=0.22, p<0.01) and anxiety (b=0.21, p<0.01). The results for the DAS-28 were in the expected direction but very small so they were not significant. These findings suggest that insomnia is related with the WSAS, but not the DAS-28, regardless of mental health status. Compared with the correlations, these findings are similar in that the correlations were larger for the WSAS than the DAS-28.

The overall mediating path was tested to determine if insomnia mediated between mental health and disease outcomes. The mediation was significant for both depression (b=0.08, p=0.03) and anxiety (b=0.07, p=0.03) for the WSAS. However, it was not significant in the mediations with the DAS-28 as the outcome. These results align with the findings that insomnia was associated with mental health and the WSAS but not the DAS-28. Thus, it appears that insomnia is a mediator between mental health and disease outcomes but only for the WSAS. The proportion mediated was 0.42 for depression and 0.15 for 0.51 for anxiety. The proportions for the DAS-28 were insignificant. Overall, it appears insomnia significantly mediates between mental health and the WSAS, with depression mediating a larger amount of the effect than anxiety.

*Alcohol*

Alcohol use was tested in the A path to determine if there was a significant association with mental health. There was not a significant association between alcohol and mental health for either depression or anxiety. The results were in the unexpected direction, but the effect size was very small and insignificant. Comparing the results with the correlations, the same unexpected direction was found, but again the size of the correlation was small.

In the B path, which tested the relationship between alcohol and disease outcomes of the WSAS and DAS-28, none of the associations were significant. The results in this B path were also in the unexpected direction, though small and insignificant. Overall, alcohol use did not appear to be significantly related with disease outcomes. Compared with the correlations, the unexpected direction remained, but the size of the correlations was also small so they did not indicate a strong relationship either.

The overall mediating path was tested to determine if alcohol use mediated between mental health and disease outcomes. The mediation path was not significant for the WSAS outcome for either the depression or anxiety models. However, the mediation for DAS-28 was significant for the depression model (b=0.21, p=0.04) but not the anxiety model. Thus, although the associations between alcohol and mental health and disease outcomes individually was not significant, alcohol does appear to mediate between depression and the DAS-28. However, the proportion mediated was very small (0.01), which is aligned with the insignificant findings for the A and B paths individually. Overall, alcohol appears to mediate between depression and the DAS-28, but the amount mediated is very small.

*Smoking*

Smoking was tested in the A path to determine if there was a significant association with mental health. There was not a significant association between smoking and mental health for either depression or anxiety. However, the effects were in the expected direction even though they were insignificant. The findings indicate that smoking does not appear to be significantly associated with mental health. Compared with the correlations, smoking and mental health had small sized correlations which align with the findings from these insignificant associations.

In the B path, which tested the relationship between smoking and disease outcomes of the WSAS and DAS-28, only the association between smoking and the DAS-28 was significant in the depression model (b=-0.11, p=0.03). However, the effect was in the same expected direction for anxiety, though it was not significant. For the WSAS, the effects were in the unexpected direction but were small and insignificant with the 95% CI including the expected inverse relationship. Thus, it appears smoking is associated with the DAS-28 when controlling for depression but not anxiety. It does not appear to be associated with the WSAS. Compared with the correlations, the correlations varied from very small to moderate but the associations from the mediation models show that only the relationship with the DAS-28 was significant when controlling for depression.

The overall mediating path, AxB, was tested to determine if smoking mediated between mental health and disease outcomes. The mediation paths for smoking were not significant in any of the mental health or disease outcome models. The proportions mediated were accordingly not significant. Overall, it does not appear that smoking mediates between mental health and disease outcomes.
